# Breakdown and Modification of Microplastic Beads by Aeolian Abrasion

**DOI:** 10.1021/acs.est.2c05396

**Published:** 2022-12-15

**Authors:** Joanna E. Bullard, Zhaoxia Zhou, Sam Davis, Shaun Fowler

**Affiliations:** † Geography and Environment, Loughborough University, Leicestershire LE11 3TU, U.K.; ‡ Loughborough Materials Characterisation Centre, Department of Materials, Loughborough University, Leicestershire LE11 3TU, U.K.

**Keywords:** wind erosion, microsphere, wear, scanning electron microscopy, fragmentation

## Abstract

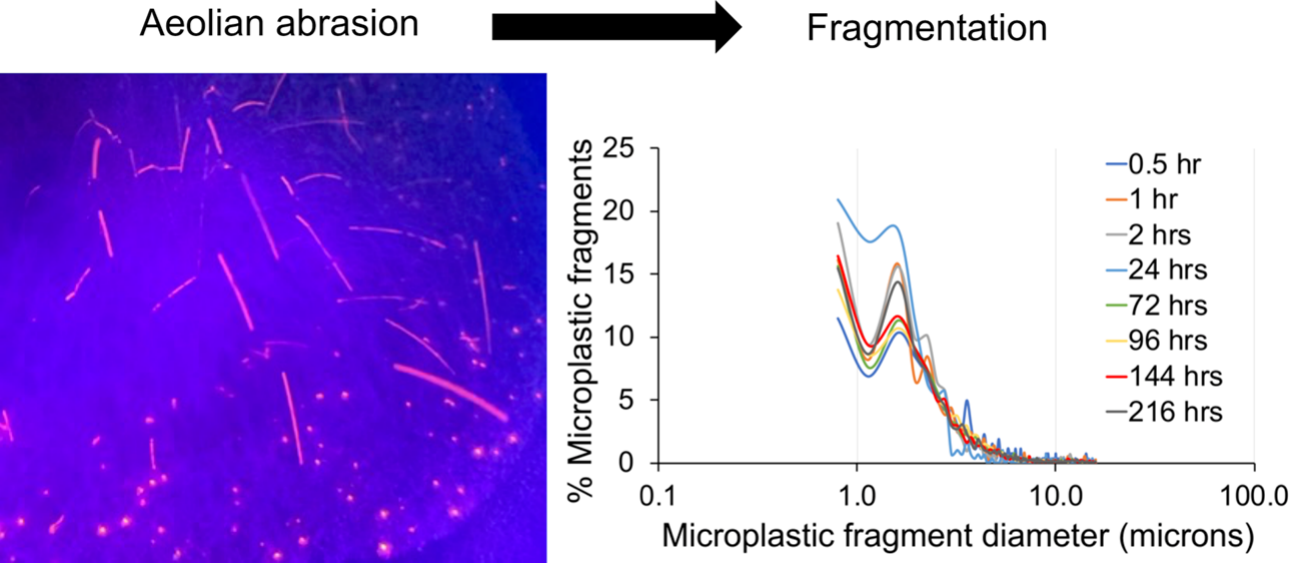

Saltation is an important wind erosion process that can cause the modification and breakdown of particles by aeolian abrasion. It is recognized that microplastic particles can be transported by wind, but the effect of saltation on microplastic properties is unknown. This study examined the impact of simulated saltation alongside quartz grains on the size, shape, and surface properties of spherical microplastic beads. The diameter of the microplastics was reduced by 30–50% over 240–300 h of abrasion with a mass loss of *c*. 80%. For abrasion periods up to 200 h, the microplastic beads remained spherical with minimal change to overall shape. Over 95% of the fragments of plastic removed from the surface of the microbeads during the abrasion process had a diameter of ≤10 μm. In addition, during the abrasion process, fine particles derived from breakdown of the quartz grains became attached to the surfaces of the microbeads resulting in a reduction in carbon and an increase in silicon detected on the particle surface. The results suggest that microplastics may be mechanically broken down during aeolian saltation and small fragments produced have the potential for long distance transport as well as being within the size range for human respiration.

## Abstract 2

Minimal research exists on microplastic breakdown during wind erosion. This study reports laboratory simulations of the aeolian abrasion of microspheres by sand with implications for microplastic surface chemistry and respiratory health.

## Introduction

Wind erosion redistributes over 6 billion tons of soil annually and is increasing in some environmentally sensitive regions.^1^ In addition to the transport of minerogenic particles, wind erosion of soils can also redistribute organic, industrial, and agricultural materials where they are present at the soil surface.^2−4^ One material found in soils and susceptible to wind erosion is plastic which has potentially serious consequences for human health, global biogeochemical cycling, and ecosystem functioning.^5,6^ Plastics have been identified in soils worldwide and can be present as both macroplastics (≥5 mm in size) and microplastics (<5 mm in size).^7^ Although the low density, high strength to weight ratio, and large surface area of some macroplastics—such as bags and balloons—mean that they can be blown considerable distances,^8^ the main research focus for wind erosion and atmospheric transport is microplastic entrainment and dispersal.^5,9^ Microplastics are solid synthetic-polymer-containing particles and may be purposefully produced to be small in size (primary microplastics) or derived from the breakdown of macroplastics (to form secondary microplastics) by chemical,^10^ microbial,^11^ and/or mechanical processes.^12,13^ Microplastics may be present in soils due to the environmental breakdown of plastic mulching material used in agriculture or the application of waste water or sewage fertilizer containing microplastics^14,15^ but have also been identified in remote or upland soils with no history of agricultural use^9,16^ where their presence is attributed to atmospheric deposition following long distance transport by wind.^16,17^

Wind erosion of sediments occurs primarily by particles moving in ballistic trajectories which may initiate the motion of surface particles via creep (rolling), dynamic saltation, or the ejection of small and/or light particles high into the airstream where they are transported in suspension. The action of wind can alter the properties of entrained particles by aeolian abrasion which includes impact collisions among minerogenic particles in the air as well as bed surface impacts. Repeated entrainment and deposition can cause chipping and spalling reducing particle size, changing the particle shape and in turn generating new, fine “silt” or “dust” particles (typically <100 μm diameter).^18−20^ The physical properties of the surfaces of minerogenic sediments can be altered during aeolian transport and may develop conchoidal fractures, upturned plates, crescentic percussion marks, linear striations, and crevasses.^21,22^ The surface chemistry of some particles may also be altered by the removal by abrasion of chemically distinct coatings.^23,24^

Wind tunnel experiments using only microplastic particles have demonstrated that they exhibit all modes of aeolian transport (creep, saltation, and suspension) but that the relationship between these modes differs from that observed in mineral particle beds.^25,26^ For example, for the same wind speed, acrylic particles (ø 192 μm, ρ 1.21 g cm^–3^) impact the surface at a smaller angle than quartz sand (ø 303 μm, ρ 2.63 g cm^–3^) and eject a greater number of particles but at lower velocities and angles than observed for quartz particles.^25^ It is unknown how impact, rebound, and ejection relationships differ between a bed comprising particles of uniform shape and density and one comprising particles with varying shapes and densities as would be expected in a mixed microplastic-sediment bed, nor how these relationships would affect the rate and nature of particle breakdown during saltation.

The mobilization of microplastic-containing soils by wind is expected to result in repeated airborne microplastic–microplastic and microplastic–mineral collisions as well as microplastic–surface impacts. Laboratory engineering experiments have shown that abrasive particles air-propelled at high speed (>50 m s^–1^) can cause erosion of immobile, solid polymers with the rate of erosion dependent on the properties of the abrasive (e.g., material, mass, and shape), the polymer type, and factors such as abrasive impact angle and velocity.^27^ The collision of the abrasive causes a range of impact marks including smoothing, plowing, cutting, cracking, grooving, and denting of the polymer.^27,28^ Cumulative impacts result in the detachment of fragments of the polymer causing mass loss. The type and depth of the impact marks and associated fragment removal can be affected by the shape of the abrasive with angular particles causing greater erosion than rounded particles.^27^ In the initial stages of erosion, an “incubation period” may occur during which there is little or no mass loss, and the polymer may actually gain mass as a result of the abrading particles becoming embedded in the sample surface.^28,29^ This embedded material can reduce the polymer erosion rate and increase particle surface roughness.^30^ The threshold wind speeds required to initiate natural wind erosion (4–8 m s^–1^) are substantially lower than the air velocities used for engineering studies of polymer breakdown (25–60 m s^–1^),^27−29^ and although high natural wind velocities have been recorded, they are rarely sustained for long periods. In addition, during wind erosion, both soil particles and microplastics are expected to be in motion affecting energy transfer processes between the particles and reducing the physical impact of the collision. Collisions and impacts are expected to affect the properties of the particles and may result in the further breakdown of microplastics to nanoplastics (≤1 μm) with implications for their distribution and environmental impact, but this has not yet been investigated within the context of wind erosion.

We conducted a series of preliminary, observational experiments that examined the impact of aeolian abrasion by quartz sand on plastic microbeads of three different sizes to determine how the size and surface characteristics of the microplastics were affected. To explore the effect of abrasive shape, an additional experiment using glass ballotini instead of quartz sand was conducted. Fragments of plastic generated during the abrasion process were examined to determine their size distribution and potential environmental implications.

## Materials and Methods

### Abrasion Experiments

**Figure 1:**
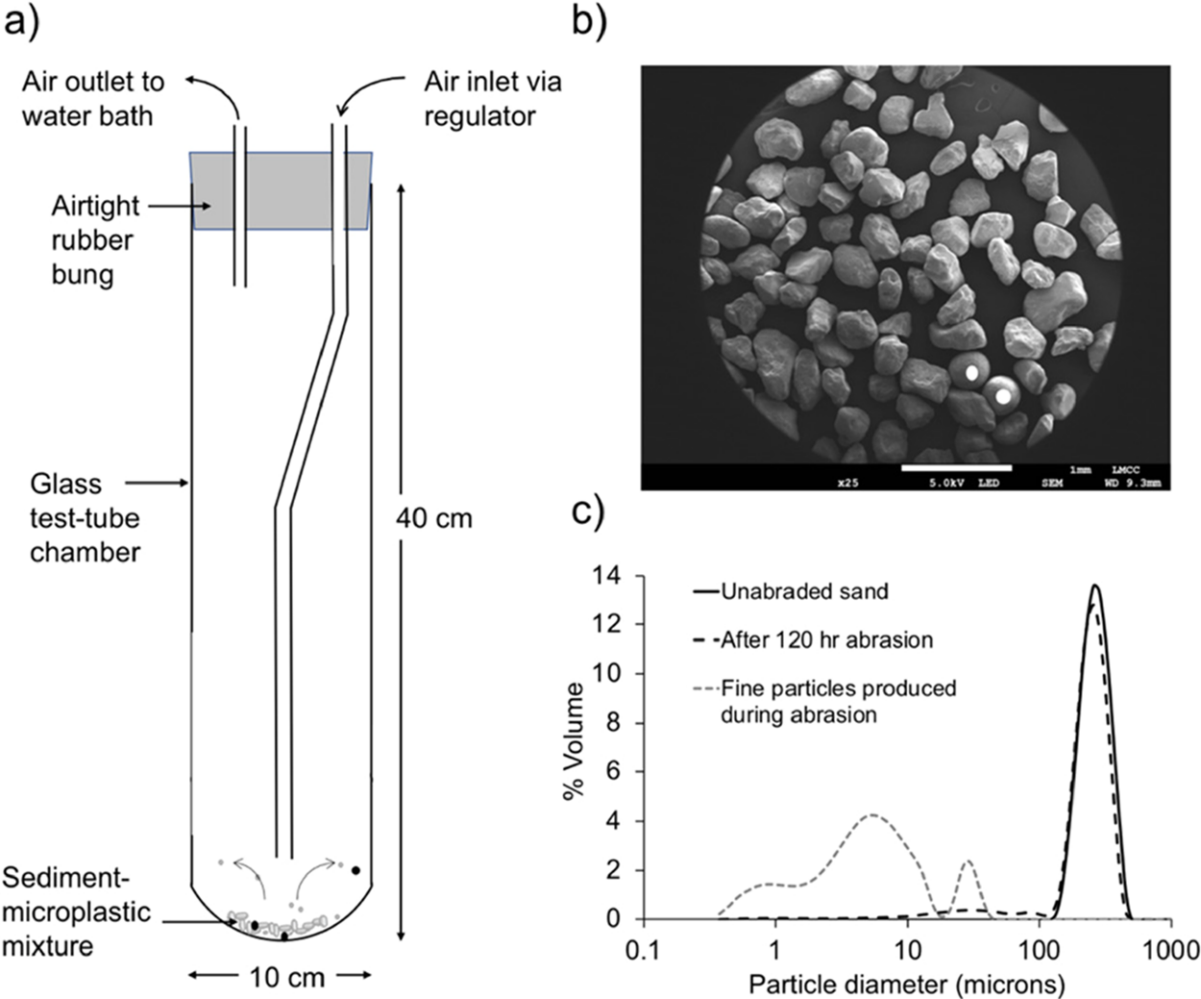
(a) Schematic diagram of the abrasion apparatus used for the experiments. (b) Scanning electron micrograph of quartz sediment used in abrasion experiments—the two particles indicated by white dots are microplastic beads. (c) Particle-size distribution of the minerogenic sediment before abrasion, after 120 h of abrasion and the fine particles produced and entering the water bath during 120 h abrasion.

A large glass “test-tube” chamber was used for the aeolian abrasion simulation experiments, based on equipment developed by Whalley et al.^31^ and Wright et al.^20^ and widely used in studies of particle breakdown and fine particle production by aeolian processes^18,32,33^ (Figure 1). The chamber enabled the simulation of continuous aeolian activity with particles creeping or rolling across the base and ejected into saltation from the centre. The sample to be abraded was placed in the bottom of the glass chamber and agitated by a constant air stream sufficient to lift particles to a height of 8–10 cm above the base of the chamber with a majority of grains rising only 5 cm. As particle motion in the chamber was continuous, it was not possible to equate abrasion rates in the chamber to abrasion rates under natural field conditions where aeolian activity is episodic. Relative humidity within the chamber was measured using an iButton under the bung and was 20–30% for all experiments to reduce capillary forces. The original chamber design incorporates a high voltage electrostatic dust trap above the glass chamber to capture fine particles lifted into suspension. To avoid electrostatic effects on the microplastics, the high voltage dust trap was removed and the interior of the glass chamber was sprayed with isopropanol and allowed to dry prior to each use to prevent the build-up of static charge. All air exiting the chamber was fed into a sealed deionized water bath to retain the particles. Any particles trapped in the air outlet tube between the glass chamber and water bath were retrieved by washing with deionized water. At the end of the abrasion period, the sample remaining in the base of the glass chamber was retained for examination using scanning electron microscopy. The contents of the water bath were filtered on to 0.45 μm cellulose acetate filter papers for further examination.

The minerogenic sediments used were washed, well-sorted, sub-rounded, sub-discoidal^34^ quartz sands with a modal diameter of 270 μm (Figure 1). The quartz sands exhibit microtextures typical of glacio-aeolian grains including bulbous edges, upturned plates, v-shaped fractures, and percussion cracks.^21^ Simulated abrasion of 10 g of the quartz sands using the apparatus described above produced 0.116 g of fine particles in 120 h in the size range 0.375 to 50 μm (Figure 1). This represents 1.062% of the initial sample weight (%ISW) which is a similar proportion to other studies of mineral sand abrasion using a comparable apparatus.^18,32^ The microplastics were fluorescent proprietary polymer (Cospheric, California, USA) microbeads comprising ≥70% polyethylene by weight with a density of 1.13–1.25 g cm^3^ in three diameter sizes: small (212–250 μm), medium (300–355 μm), and large (500–600 μm). Polyethylene was used because this is one of the most common polymer types found in surface soils (0–10 cm).^35,36^ Each experiment used *c*. 10 g of sediment and 0.01 g of microplastic, i.e., a microplastic concentration of 0.1% which is at the upper end of the concentrations found in agricultural soils.^35^ Microplastic particle counts were 1549, 544, and 115 per 0.01 g for small, medium, and large microbeads which gives ratios of microbeads to quartz grains of approximately 1:236, 1:673, and 1:3185, respectively. To provide an indication of the role of erodent shape in microplastic abrasion, an additional set of experiments was conducted using the medium-sized microbeads and smooth-surfaced, spherical borosilicate glass ballotini (500–700 μm diameter; density 2.23 g cm^3^) as the erodent, rather than the quartz sand.

Experiments using minerogenic sediments have shown that the mass, particle-size characteristics, and surface properties of parent and derived sediments change according to the duration of abrasion.^18,23^ For this study, discrete experiments were run for each abrasion period and ranged from 0.5 to 330 h.

### Particle Characterization

For the minerogenic sediments, the particle size distribution was determined using a Beckman-Coulter LS280 laser-sizer in the range 0.375–2000 μm with 92 class intervals.

For the experiments using quartz sand and medium-sized microbeads, microplastic fragments produced during the abrasion process and captured on the filter papers were examined using fluorescence microscopy. The medium microplastics and fragments derived from their breakdown are bright blue with a strong fluorescence response. Fluorescence excitation wavelengths were between 250 and 350 nm, and images of the filter papers were captured at a magnification of ×200 using a Leica DMRX compound microscope with a high-pressure mercury UV lamp source. Images were analyzed using ImageJ by applying thresholding to isolate blue particles. Blue particles straddling the image edge were excluded. Very large irregular blue shapes were also excluded from analysis as these were most likely aggregated particles and represented a negligible percentage of the dataset. As the abrasion chamber had been modified from its original design, the efficiency of capture of particles in the outgoing airstream is unknown. Where considerable quantities of fine minerogenic particles (“dust”) were produced, microplastic fragments may have been obscured by these on the filter papers. The number and mass of microplastic particles retrieved were very small, and it was not possible accurately to physically separate them from the minerogenic fine particles to determine the mass of microplastics in atmospheric suspension compared to the initial sediment sample weight. For this reason, the size distribution of microplastic fragments is expressed as % of particles retrieved (as opposed to the conventional %ISW).

The total mass of material eroded from the microplastics with the quartz abrasive was estimated using the mean microbead diameter after each period of abrasion, density of the microplastic, and initial microbead particle count per 0.01 g.

### Scanning Electron Microscopy

For all experiments (quartz/glass + all sizes of microplastic), the microbeads remaining in the glass chamber after abrasion were examined using scanning electron microscopy (JSM-7800F field emission scanning electron microscope: SEM) with 5 kV electron accelerating voltage. All samples were coated with gold/palladium before the analysis to limit surface charging. Using SEM, the dimensions of the microbeads were measured and close-up micrographs of the bead surfaces were captured to visualize any changes. Energy-dispersive spectroscopy (EDS) was used to map the surface element composition of microplastics after each abrasion experiment.

For the quartz sand with medium-sized microbead experiments, additional analyses were conducted to provide more detailed information about surface and subsurface changes to the microplastics. The surface of the microbeads was micro-milled using a Dual Beam System Focused Ion Beam SEM (FIB-SEM: FEI-Nova600 Nanolab Ga Dualbeam) to determine the surface and subsurface composition of the particles. An area of the microbead surface *c*. 10 × 2 μm was coated with platinum before bulk milling a trench, after which the surface was cleaned using low current to leave a damage-free cross-section. The surface and subsurface section was then scanned using EDS. The depth of any surface changes observed was determined by taking 3–5 measurements within the section.

## Results and Discussion

### Microbead Size and Shape

**Figure 2:**
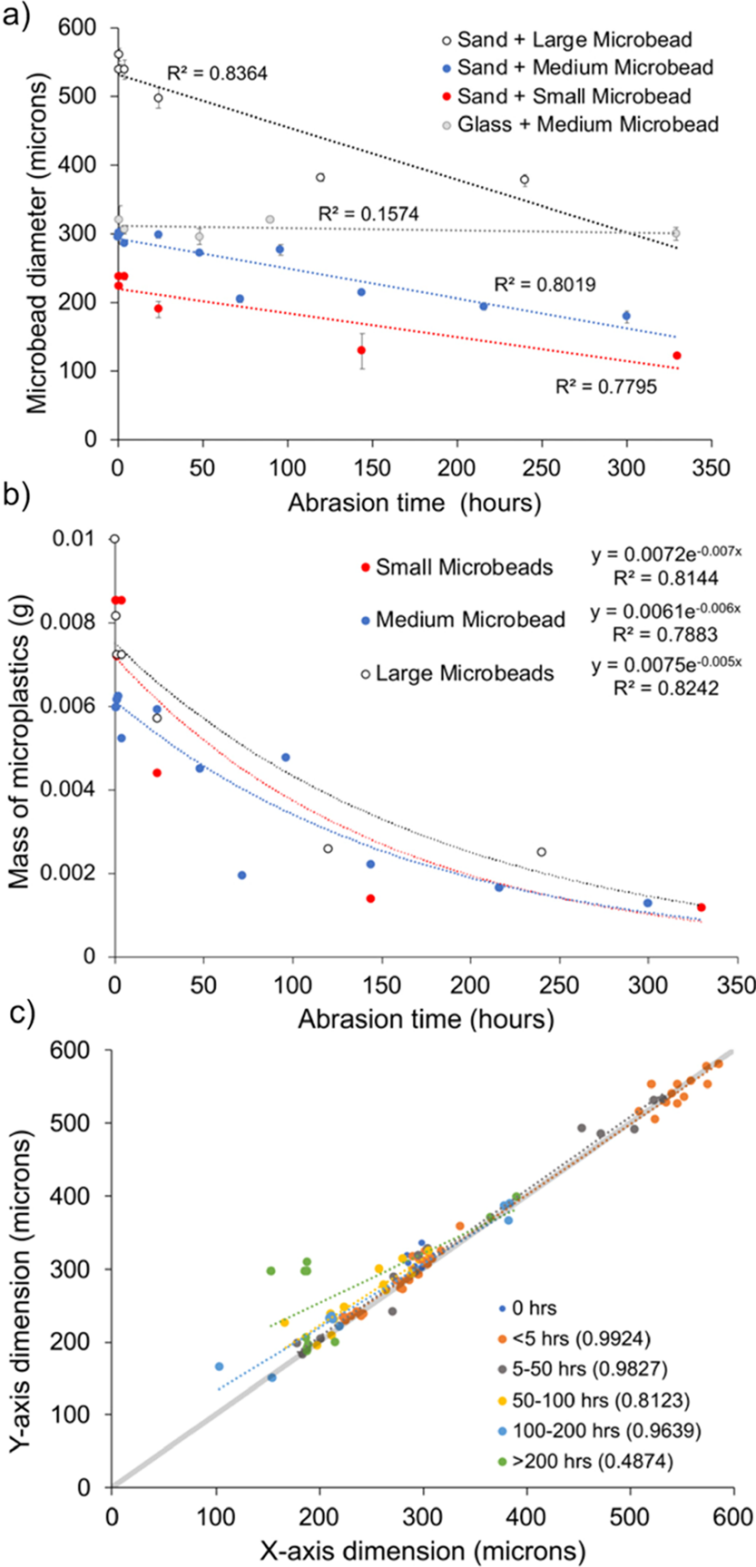
(a) Change in the microbead diameter after different durations of abrasion. Values are average diameters for measured beads +/– 1 standard error. (b) Estimated mass of microplastics remaining in the abrasion chamber. (c) Relationship between *X*-axis dimension and *Y*-axis dimension for all microbeads measured using SEM and grouped by duration of abrasion (hours). *R*^2^ values for the relationship between *X* and *Y* measurements given for each group in brackets in the legend. The pale gray solid line indicates a 1:1 relationship.

The erosion, or wear, rate of a material depends on the number and mass of the individual particles striking the surface and their impact velocity.^29^ For the experiments reported here, air pressure, and hence impact velocity, remained constant but abrasion time was varied and longer abrasion periods will increase the number of particle strikes. Consistent with this, we observed a decrease in the microbead diameter with longer periods of wear where the erodent was quartz, but this was not seen in the experiments using glass ballotini (Figure 2a). In all cases, there is a statistically significant negative relationship between microbead size and abrasion time when the microplastics are abraded with natural quartz sand (*p* ≤ 0.01 medium/large; *p* ≤ 0.05 small). The change in the diameter of the microplastics is greater than that previously observed for quartz particles. For example, Bullard et al.^18^ reported a reduction in the modal particle diameter of quartz sand from 265 to 252 μm and 190 to 115 μm over 120 h (reduction of 13 and 75 μm respectively), whereas the small microplastic beads were reduced from 250 to 190 μm in 24 h and then to 129 μm in 144 h (reduction of 108 μm).

The relative hardness of interacting particles influences the rate of wear. The hardness of the glass is >5000 HV, the sand *c*. 1200 HV, and polyethylene 10 HV.^37,38^ This suggests that glass particles would be more abrasive than sand; however, when the microplastics were abraded with glass ballotini, there was no significant change in the microbead diameter over 330 h (Figure 2a). A key factor in the effectiveness of an abrasive is particle shape (comprising form, roundness, and roughness). Walley & Field^27^ found that for the same impact velocity, sand grains caused more erosion and surface damage to polyethylene sheets than steel spheres and attributed it to the angularity of the natural particles providing denser contact points. The difference in the wear rate between angular and rounded particles can be a factor of 10 or more.^38^ Rounded particles can deform the surface by plowing, displacing materials to the side and in front of the impact, and angular particles are more likely to cut or indent the surface. Plowing, displacement, and indentation deformation signatures were all observed using SEM where the microplastics had been abraded by sand and the roughness microtextures (such as “edges”; Figure 1) on the abrasive quartz particles are likely to have contributed to this deformation and wear. The spherical glass ballotini had very smooth surfaces with minimal microtexture compared to the sand, and the use of a fixed, rather than variable, airflow into the abrasion chamber means that the coarse ballotini underwent less movement and had lower impact velocities than the finer sand particles. These factors likely contributed to the lack of erosion of the microbeads abraded with the ballotini.

Taking in to account the total number of microplastic beads included in each experiment, the estimated total mass loss of microplastics was similar for all sizes when the quartz abrasive was used (Figure 2b). Assuming that the change in the diameter and consequently volume of the microbeads is due to erosion rather than compression, the estimated mass of microplastic beads (all sizes) remaining in the abrasion chamber after abrasion with quartz sand is *c*. 0.002 g which is 20% of that at the start of the experiment. At the individual particle scale, over the first 24 h on average each large microbead lost 0.037 mg which is an order of magnitude more than those of the small and medium microbeads, which eroded by 0.0036 and 0.0075 mg, respectively, over the same time period. Some previous studies of quartz sand abrasion have found a positive relationship between the grain size and abrasion rate,^19^ but this is not universal,^32^ and controls such as particle shape and sorting are likely to be at least as important. Abrasion rates are expected to vary according to the number of active particles in the abrasion chamber and probability of collisions taking place.^32^ As the samples are measured by weight, there are more small microplastic beads per 0.01 g (1549) compared to large microplastic beads (115) so the probability of a small microbead colliding with a quartz grain is higher, but the amount of mass loss per collision may be smaller.

For all microbeads, abrasion for up to 200 h duration appears to have very little impact on microbead shape such that the *X* and *Y* dimensions of the microbeads plot as a near 1:1 relationship (Figure 2c). This suggests that wear within the aeolian abrasion chamber occurs evenly. For periods of abrasion longer than 200 h, there is some indication that abrasion might cause microbeads to become less spherical, but a greater number of tests and longer periods of abrasion are needed to confirm this.

### Microplastic Fragmentation

**Figure 3:**
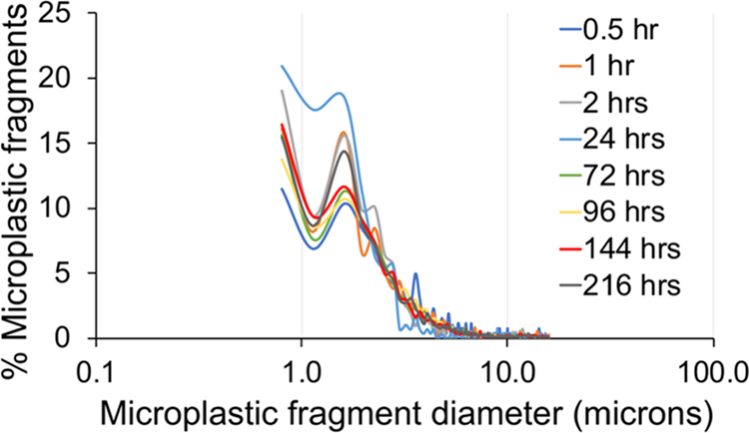
Size distribution of microplastic fragments retrieved following different periods of abrasion of the medium-sized microplastic beads.

Fluorescence microscope analysis of microplastic fragments created during the abrasion process revealed that the particle-size distribution of the fragments was similar for all durations of abrasion with a clear mode in the 1–4 μm range and a probable finer mode <1 μm (Figure 3). The image analysis technique used could not fully represent particles smaller than 1 μm.

More than 95% of the eroded microplastic fragments trapped and analyzed were ≤10 μm diameter and are therefore respirable by humans. For abrasion periods ≥1 h, more than 54% of the fragments were ≤2.5 μm and 24–38% were ≤1 μm and therefore inhalable. The impact of microplastic inhalation by humans varies with both their inherent physical (size and shape) and chemical properties and also their ability to act as a vector for microorganisms, toxins, and persistent organic pollutants following environmental exposure.^39−41^ Very little empirical research into the effects of microplastics on respiratory human health has been carried out; however, research on other airborne microparticles suggests that small respirable particles are more harmful to human health than larger particles because they can enter the lungs and interact with cells and tissues. In addition, the effective surface area of particles is important as greater surface roughness increases interactions between microparticles and cells.^42^

### Microbead Physical and Chemical Surface Characteristics

**Figure 4:**
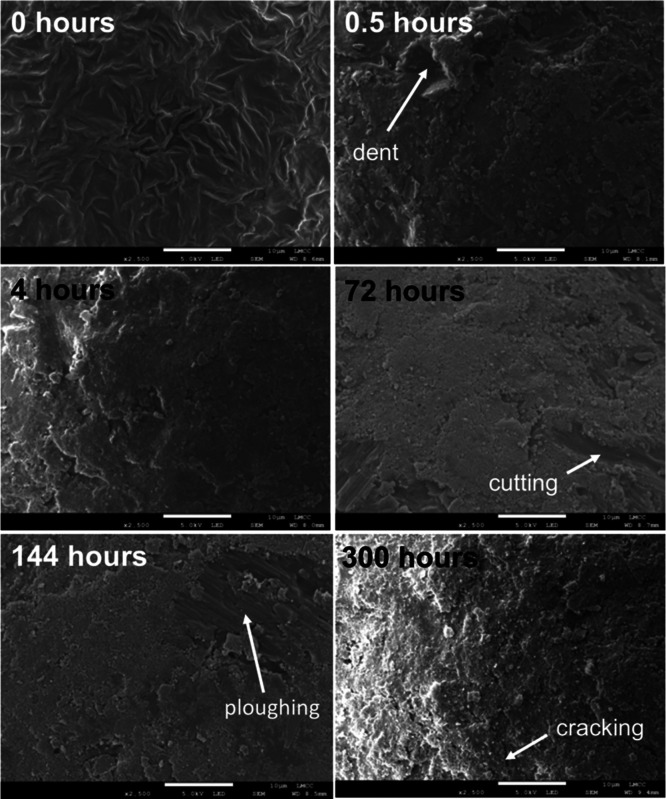
Scanning electron micrographs of medium-sized microbead surfaces after selected periods of abrasion. For all images, the white scale bar is 10 μm.

The physical and chemical surface properties of the microbeads were examined using SEM and underwent fundamental alterations when sand was used as an abrasive (Figure 4). Unabraded beads had an evenly distributed labyrinthine pattern on the plastic surface (0 h). After 0.5 h of abrasion with sand, the surface had visibly changed. Small particles of the quartz abrasive were visible on the surface of the microbeads and embedded in, or otherwise attached to the surface of the plastic, for example by tribocharging or van der Waals forces.^43,44^ Where the microplastic surface was still visible, wear patterns including smoothing, plowing, cutting, and cracking developed. Cracks were primarily “switch” (bifurcating) and curve types.^45^ The unabraded sand did not contain any particles of <100 μm diameter, but abrasion of the sand resulted in production of fine particles in three modes at 0.5–1 μm, 5–6 μm, and *c*.30 μm (Figure 1). SEM analysis of the quartz mineral particles attached to the microplastic beads indicates that they were predominantly in the range 0.2–1 μm which suggests that they were derived from abrasion of the sand.

**Figure 5:**
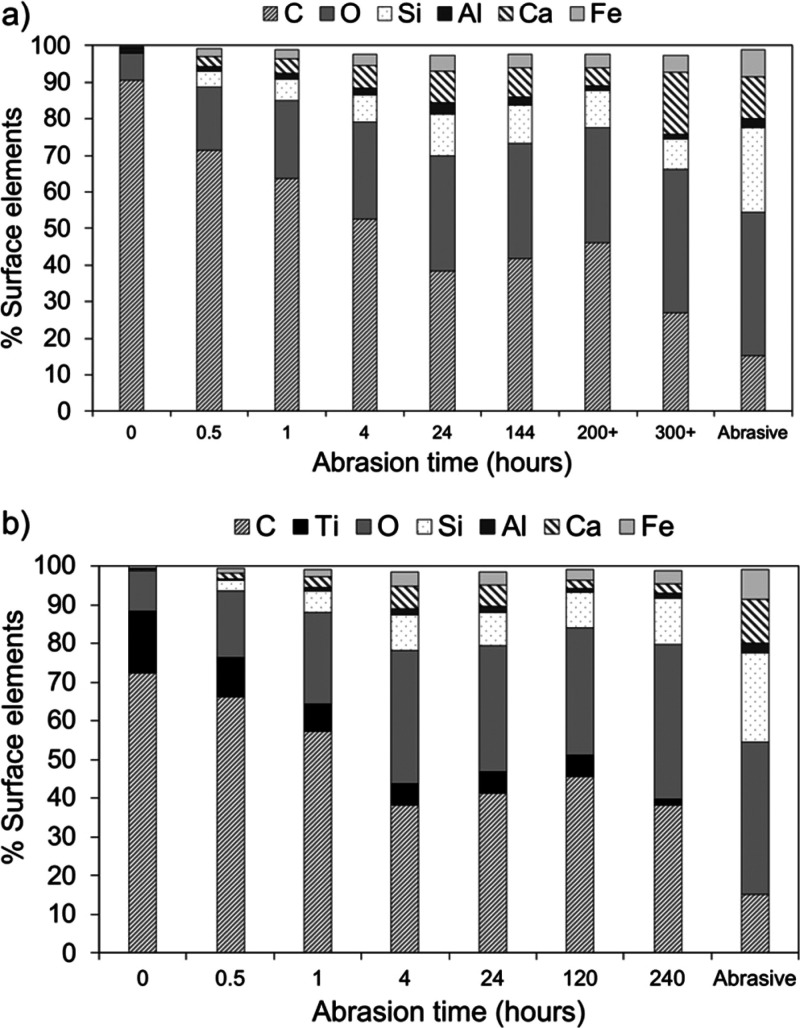
Surface elements present on the surface of (a) small/medium microbeads and (b) large microbeads. The surface composition of the abrasive sand with no abrasion is also shown.

Full results of the EDS analyses are given in the Supporting Information (Tables S1–S4). The small and medium-sized microplastics had similar surface compositions prior to abrasion where the dominant element was carbon (85.3–95.9%) with small percentages of oxygen (3–11%), aluminum (<4%), and iron (<0.5%) (Figure 5a). The large microplastic beads were white, and 15.8% of the surface composition was titanium (Figure 5b). Other trace elements detected were sodium (<0.5%), magnesium (<0.3%), sulfur (<1.2%), and potassium (<1.5%). Following >300 h of abrasion, the % of carbon detected on the microplastic surfaces decreased from >80 to <30% for small and medium microbeads. For large microbeads, the % carbon decreased from >70 to <40% after 240 h. With more abrasion, the microplastic surfaces gain oxygen and silicon. The large microbeads showed a steady decline in the % titanium detected from 15.8% (0 h abrasion) to 1.6% (after 240 h abrasion). EDS analysis of a sample of 10 of the quartz sand particles used as the abrasive is also given in Figure 5. The % aluminum on the microbeads remained low throughout (<4.2%) with no systematic gain/loss during abrasion and was similar to that of the abrasive (2.6%). Calcium was not present on the unabraded microbeads but was present following all periods of abrasion but very variable from 3.9 to 26.3% on the small microbeads, 1.9 to 7.4% on the medium microbeads, and 1.3 to 5.7% on the large microbeads. The abrasive particles included 0 to 37.9% calcium which may indicate the presence of shell fragments that were transferred to the microplastics.

**Figure 6:**
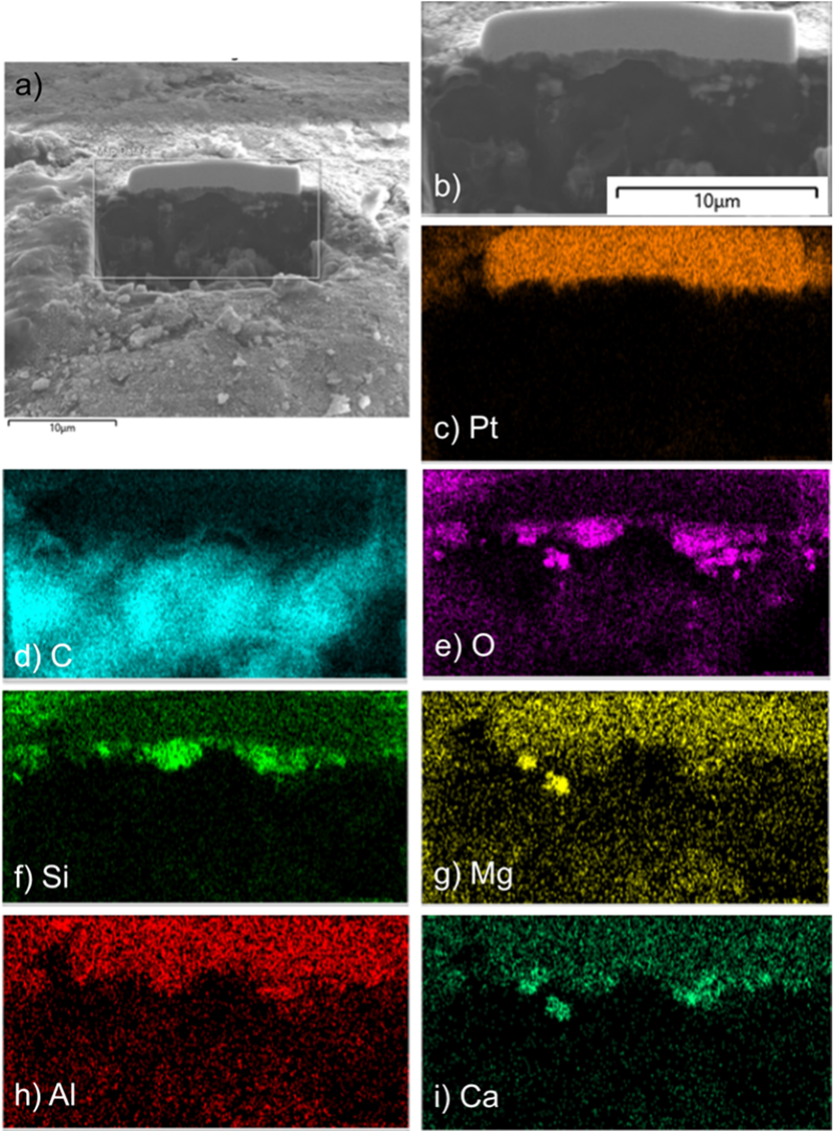
(a) Scanning electron micrograph of the surface of the medium-sized microplastic bead after 144 h of abrasion showing micromilling of the bead using a focused ion bean (FIB) to enable determination of the depth and composition of the surface coating. (b) Close-up view of the excavated profile and results of energy-dispersive X-ray spectroscopy (EDS) showing the presence of (c) platinum, (d) carbon, (e) oxygen, (f) silicon, (g) magnesium, (h) aluminum, and (i) calcium.

**Figure 7:**
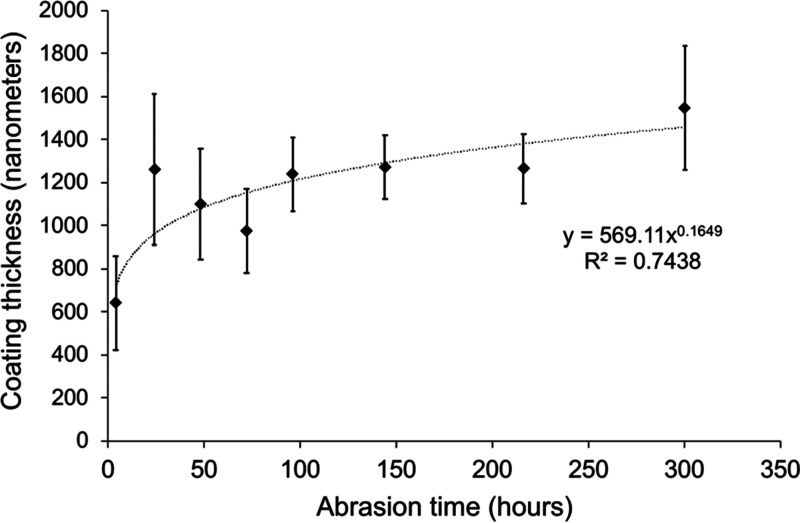
Thickness of coating developed on medium (300–355 μm) microbeads following different periods of abrasion.

EDS mapping of the exposed cross-sections of the medium-sized microbeads showed that the core of the microbead was primarily carbon and a thin layer of mineral grains, detectable from the presence of oxygen and silicon, was present at the surface (Figure 6). The mean thickness of the mineral grain coating after 4 h of abrasion was 640 nm and developed rapidly to a thickness of 1000–1500 nm (1–1.5 μm) after 24 h (Figure 7). This thickness was consistent with a single layer of the smallest minerogenic particles produced by abrasion of the quartz sand. During abrasion, microbead diameters decreased, but the thickness of the minerogenic coating did not substantially change after the first 24 h. A possible explanation for this is that the mineral particles become embedded in the microplastic but are then dislodged by repeated impacts. When dislodged, the mineral particles remove some of the plastic gradually reducing the diameter of the bead but are then replaced by more particles but only to a thickness of one grain.

**Figure 8:**
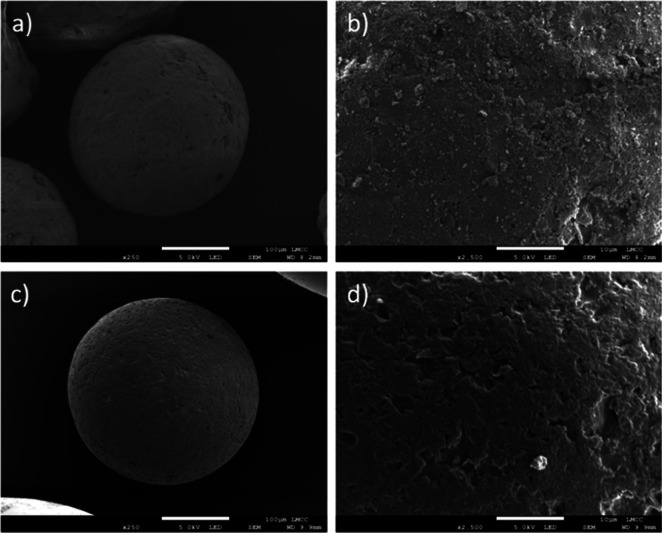
SEM of the medium-sized microbead (white scale bar 100 μm) and close-up of the microbead surface (scale bar 10 μm) following (a) and (b) 96 h of abrasion with sand; (c) and (d) 90 h of abrasion with glass ballotini.

For the microplastics abraded with glass ballotini, the physical surface texture of the bead was altered but retained elements of the pre-abrasion labyrinthine patterns (Figure 8). Abrasion using glass ballotini did not result in any appreciable change to the surface composition of medium-sized microbeads which after 90 h of abrasion comprised 83.6% carbon, 12.4% oxygen, and 1.4% silica. This is more similar to unabraded microbeads (95% carbon, 3.4% oxygen, and 0% silicon) than those abraded with sand for 96 h (43.6% carbon, 34.6% oxygen, and 10.8% silicon).

### Implications for Microplastic Breakdown by Wind Erosion

A limitation of most existing aeolian sediment transport equations is that they assume uniform particle shape (usually spheres) and density.^46^ It is expected that the relationships among the primary modes of aeolian transport will be altered by the incorporation in a sediment bed of microplastics with a lower density than the mineral particles.^25^ This is because the momentum transfer that occurs during saltation impact will differ with particle density. Low density microplastics may have insufficient momentum to eject mineral particles from the bed surface, whereas higher density mineral particles may preferentially cause the ejection of microplastics into the atmosphere.^47,48^ In addition, microplastic shape is expected to influence the transfer of momentum from dynamic to static particles where compact microplastics are likely to be more effective than pliant (fibrous) microplastics, but this has yet to be investigated in the context of wind erosion for interacting particles of substantially different shapes and densities. Whether or not the presence of microplastics in soils will significantly influence wind erosion processes is likely to depend on the concentration of microplastics and how they interact with the mineral component, and this remains a priority for future research.^49^

There are ongoing debates around the relevance of microplastic experiments using commercially produced, pristine microplastics (as used here) compared to primary or secondary microplastics harvested from the natural environment^50,51^ but clear recognition that controlled experiments isolating the impact of different variables are valuable.^52^ Pristine microplastics were used here to ensure comparability among experiments in terms of the material, size, and shape of microbeads. The microplastics used had not been exposed to natural environmental conditions, such as exposure to solar radiation, which is expected to increase brittleness and hence susceptibility to fragmentation.^13^ Microplastics are diverse with varying shapes, densities, and sizes. The experiments reported here used spherical, compact microbeads. Microbeads can be present in soils as a result of treatments such as the application of biosolids for fertilization^7,14^ but are not as common as films derived from plastic film mulching or fibers from long distance atmospheric fallout.^6,53,54^ For mineral particles, processes of entrainment, transport, and interaction are known to vary with particle shape, and because the same will be true for microplastic particles, the abrasion processes reported here are only expected to be directly relevant to compact microplastics. Microplastic fibers and fragments can exhibit weathering and wear patterns similar to those reported here, including the attachment of small mineral particles to the polymer surface,^55^ and further controlled experiments are required to determine how rapidly such features develop on microplastics of different shapes during aeolian abrasion.

Despite the limits to the complexity that is experimentally attainable in this type of study, our results clearly indicate the potential for microplastic fragmentation by aeolian abrasion. Importantly, we have demonstrated that the microplastics were eroded, reducing their overall size and generating fine particles with the potential for long distance transport by suspension. In addition, we have determined that the surface characteristics of the microplastics can be physically and chemically changed during abrasion as fine mineral particles become embedded in the plastic. This changes the surface roughness of the microbeads and may have implications for their ability to carry organic pollutants and heavy metals.^55^ Research into the interaction of biological organisms and polymer surfaces has shown that they can initiate or promote microplastic degradation, but conversely, once accumulated, biofilms can also protect polymer surfaces from degradation by shielding them from UV radiation.^56^ Similarly, the repeated attachment and removal of fine mineral particles to the polymer surfaces appear to result in erosion of the microplastics, but in the natural environment, such a mineral coating may provide some protection from chemical degradation, including by UV radiation, and this remains to be researched.

## Supporting Information Available

Full results of the EDS analyses including trace elements separated by size and for each individual time period (xlsx)
